# Safety Evaluation of Carbon Dots in UM-UC-5 and A549 Cells for Biomedical Applications

**DOI:** 10.3390/cancers16193332

**Published:** 2024-09-29

**Authors:** Carla M. Magalhães, Eduarda Ribeiro, Sónia Fernandes, Joaquim Esteves da Silva, Nuno Vale, Luís Pinto da Silva

**Affiliations:** 1 Chemistry Research Unit (CIQUP), Institute of Molecular Sciences, Department of Geosciences, Environment, and Spatial Plannings, Faculty of Sciences, University of Porto, Rua do Campo Alegre s/n, 4169-007 Porto, Portugal; up201201533@fc.up.pt (C.M.M.); up201708297@fc.up.pt (S.F.); jcsilva@fc.up.pt (J.E.d.S.); 2 PerMed Research Group, Center for Health Technology and Services Research (CINTESIS), Rua Doutor Plácido da Costa, 4200-450 Porto, Portugal; eduardaprr@gmail.com; 3 CINTESIS@RISE, Faculty of Medicine, University of Porto, Alameda Hernâni Monteiro, 4200-319 Porto, Portugal; 4 Department of Community Medicine, Health Information and Decision (MEDCIDS), Faculty of Medicine, University of Porto, Rua Doutor Plácido da Costa, 4200-450 Porto, Portugal

**Keywords:** nanomaterials, carbon dots, bioimaging, drug delivery, cancer cells, anticancer activity

## Abstract

**Simple Summary:**

Carbon dots (CDs) are carbon-based nanomaterials with versatile applications, including fluorescence imaging, drug and gene transport, drug delivery, medical diagnosis, and biosensing. In this study, we successfully synthesized various CDs without significantly impacting the cell viability of cancer cells, which suggests the potential for future bioimaging and drug delivery applications. These findings contribute to advancing the potential of CDs in various biomedical research contexts.

**Abstract:**

Backgroung: The rising complexity and associated side effects of cancer treatments highlight the need for safer and more effective therapeutic agents. Carbon-based nanomaterials such as CDs have been gaining prominence for their unique characteristics, opening avenues for diverse applications such as fluorescence imaging, drug and gene transport, controlled drug delivery, medical diagnosis, and biosensing. Despite promising advancements in research, it remains imperative to scrutinize the properties and potential cytotoxicity of newly developed CDs, ensuring their viability for these applications. Methods: We synthesized four N-doped CDs through a hydrothermal method. Cell viability assays were conducted on A549 and UM-UC-5 cancer cells at a range of concentrations and incubation times, both individually and with the chemotherapeutic agent 5-fluorouracil (5-FU). Results: The obtained results suggest that the newly developed CDs exhibit suitability for applications such as bioimaging, as no significant impact on cell viability was observed for CDs alone.

## 1. Introduction

Cancer poses a formidable challenge due to the complexity of available treatments and associated side effects. In 2020 alone, there were 18.1 million reported cases worldwide, resulting in 10 million deaths [1]. Numerous research groups have been dedicated to advancing therapies, such as targeted therapy and immunotherapy, as well as developing novel chemotherapeutic agents [2,3,4]. Despite these efforts, the limitations of targeted therapies, which only address specific targets, and the occurrence of severe side effects, including skin reactions and autoimmune responses, contribute to suboptimal and inefficient cancer treatments for patients [5,6,7]. Hence, there is a crucial need for developing safer and more effective therapeutic agents, particularly those capable of precise delivery to the desired target site. Over the past few decades, nanotechnology has emerged as a dominant force in cutting-edge research across different fields, including biomedicine, molecular diagnostics, pharmaceuticals, optoelectronics, and environmental preservation [8,9,10,11,12,13]. The prominently developed components involve nanoparticles, such as liposomes, solid lipid nanoparticles, dendrimers, polymers, and materials like silicon and carbon, characterized by sizes smaller than 100 nm [14,15,16,17,18].

Lately, significant focus has been directed towards carbon-based nanomaterials, including Carbon Dots (CDs), fullerenes, Carbon nanotubes (CNTs), and Graphene Quantum Dots (GQDs) [19,20,21,22]. CDs stand out as a type of carbon nanomaterial with sizes below 10 nm [23,24] and with a core that is thought to be composed of sp^2^ carbon connected by sp^3^ carbon atoms in between [25,26,27]. On their surface, different types of functional groups (such as carboxylic acids, alcohols, and amines) can be found whose identity depends on the type of precursor used and the synthesis route employed [28,29]. These nanomaterials boast unique properties such as high photoluminescence, cost-effective synthesis, chemical stability, and environmental friendliness. These distinctive properties enable diverse applications, including imaging, drug and gene transport through conjugation, controlled drug delivery, medical diagnosis, and biosensing [25,26,27,28,29]. CDs can be prepared through “top-down” and “bottom-up” strategies [11,25,28,30]. Top-down synthesis involves the breakdown of large carbon structures into smaller ones [27,30,31]. However, bottom-up synthesis is the more commonly applied method, where CDs are produced from smaller carbon structures (such as carbohydrates and citric acid) through processes like calcination, hydrothermal treatment, and microwave-assisted synthesis [30,32,33]. These pathways are often favored because they are a cost-effective, environmentally friendly, and non-toxic approach for CD production [34,35,36].

Citric acid emerges as the preferred precursor for CD synthesis due to its affordability, ready availability, and low carbonization temperature [16,28,32]. Nonetheless, using citric acid in the synthesis process tends to result in low photoluminescence [37,38]. Thus, to overcome this problem, heteroatom-doping of CDs (using N, S, P, and B) is employed to enhance the fluorescence quantum yield (QY_FL_) [11,16,39]. The most commonly employed strategy is N-doping, where a nitrogen-rich molecule, such as urea [34,40,41] or ethylenediamine (EDA) [41,42], is added to the carbon precursor. Furthermore, an additional nitrogen atom offers five valence electrons for bonding with carbon atoms [43,44]. Indeed, when employing bottom-up strategies, a diverse range of precursors can be used, enabling the production of CDs without the need for sophisticated equipment [32,45]. CDs can be generated using various biomass or organic waste as precursors, facilitating the valorization of different residues within a circular economy framework [46,47].

Given the exponential development and study of new CDs, it is crucial to evaluate their biocompatibility to assess their potential applications and guide further application studies. Numerous researchers have been synthesizing CDs from several natural sources and investigating their applicability in cancer treatment and bioimaging. For instance, Li et al. extracted fluorescent CDs from ginger and tested them on different cancer cell lines, including the human lung cancer cell line (A549), human breast cancer cell line (MDA-MB-231), human cervical cancer cell line (HeLa), and human hepatocellular carcinoma cell line (HepG2). Their findings revealed a higher growth suppression effect in HepG2 cells [48]. In another study, Xie et al. used date pits to synthesize CDs and explored their impact on the growth of cancer cells, including A549 cancer cells, breast cancer (MCF-7), and prostate cancer (PC3) cells. The inhibition rates were reported as 52%, 65%, and 26%, respectively [49]. Additionally, Wang et al. combined Metformin (Met) and CDs for integrated tumor imaging and therapy, demonstrating a significant inhibitory effect on A549 cancer cells [50].

Despite these promising advancements and positive outcomes in existing research, it remains crucial to investigate the properties and potential cytotoxicity effects of newly developed CDs to determine their suitability for in vivo/vitro imaging and drug delivery applications. Hence, this study aims to assess the chemical properties and the biocompatibility of four N-doped CDs in A549 and UM-UC-5 cancer cell lines. To that end, these CDs were prepared through a hydrothermal approach based on routes previously documented by our research group [32,51]. Then, an MTT assay was performed to evaluate the cytotoxicity of CDs in A549 and UM-UC-5 cancer cell lines. Moreover, to gain additional insights into the potential application of these CDs in drug delivery, we explored their interactions in the presence of an anticancer drug, 5-Fluorouracil (5-FU) [52,53].

## 2. Materials and Methods

### 2.1. Synthesis of CDs

In this study, four CDs were synthesized, following previously described procedures [52,54]. The synthesis followed a hydrothermal methodology. In brief, for the synthesis of CD-1, CD-2, and CD-3, 0.075 g of citric acid (CA) and 0.075 g of corn stover (whereas for CD-4, 0.150 g of CA were used) were combined with ethylenediamine (EDA, 166.9 µL) and dissolved in 0.01 M NaOH (10 mL). This mixture was placed in a Teflon-lined container, then enclosed in a steel outer shell. The reaction proceeded for 4 h (CD-1), 16 h (CD-2), or 24 h (CD-3) at 200 °C in an oven (VWR DL 112 Prime oven, 2500 W power consumption, Avantor, PA, USA). Following this period, the container was allowed to cool to room temperature, and the reaction products underwent centrifugation (6000 rpm, 30 min) to eliminate insoluble components. Subsequently, dialysis was performed (24 h, molecular weight cutoff of 3.5 kDa) to remove soluble by-products formed during the synthesis that could impact the properties and photoreactivity of the nanoparticles [13]. Finally, to determine the mass concentration of the CDs, the purified CD solution underwent lyophilization, was weighed, and dissolved in deionized water (5 mL). The CDs were then stored in cold conditions (4 °C) until use. The synthesis yields obtained were 8.4%, 6.2%, 5.5%, and 2.4% for CD-1, CD-2, CD-3, and CD-4, respectively.

### 2.2. Characterization of CDs

AFM analysis was conducted by using the tapping mode with a Veeco Metrology Multimode/Nanoscope IVA. For this analysis, a silica plate was used to deposit the sample for analysis, and an AFM TESP-SS cantilever (curvature radius of 2 nm) was used. Samples were prepared via drop evaporation of the diluted CD solution, and NanoScope v. 1.40 was the software used for the AFM data analysis. Particle size measurements were made based on the height of the particles, and the average size was calculated by averaging the measurements of 100 individual particles. The absorption spectra were recorded using a VWR^®^ UV3100PC spectrophotometer (with the UV–Vis Analyst software v.5.44) and standard 10 mm quartz cells. The fluorescence spectra of the CDs were obtained using a Horiba Jovin Yvon Fluoromax-4 spectrofluorimeter using the FluorEssence software v.3.9 to analyze the results. Slit widths of 5 nm or 2 nm were employed when obtaining 2D matrices and fluorescence spectra emission, respectively. Photostability was evaluated by exposing the CD sample to a continuous LED light source (OceanOptics, Orlando, FL, USA) of 365 nm. Then, fluorescence spectra were obtained at t = 10, 15, 20, and 30 min. Standard 10 mm fluorescence quartz cells were used. The Zeta Potential was measured by using a particle analyzer Anton Paar LitesizerTM 500 (Anton Paar, Graz, Austria) and a polycarbonate Omega Cuvette (Ref. 155765).

### 2.3. Fluorescence Quantum Yield (QY_PL_) Calculations

The fluorescence quantum yield (*QY_FL_*) was determined using a standard procedure that involved comparing the integrated photoluminescence intensities and absorbance values of the prepared CDs with those of a reference fluorophore. The *QY_FL_* value, expressed as a percentage, is calculated using the following equation:

$${QY}_{FL}^{Sample}={QY}_{FL}^{Ref}\times \frac{{Grad}_{Sample}}{{Grad}_{Ref}}\times \frac{{\eta_{Sample}}^{2}}{{\eta_{Ref}}^{2}}\times 100$$

In the above equation, Grad corresponds to the gradient of the plot of integrated fluorescence intensity versus absorbance for both the sample and the reference fluorophore and η is the refractive index of the medium. Quinine sulphate was chosen as the reference fluorophore (*QYFL* = 54%) for the quantum yield calculations due to the similarities of its photoluminescent properties to those of our CDs [54].

### 2.4. CDs Toxicity Assays

#### 2.4.1. Cells, Medium, and Compounds

A549 adenocarcinoma human alveolar basal epithelial cancer cell and UM-UC-5 urothelial bladder cancer cell lines were obtained from the American Type Culture Collection (ATCC; Manassas, VA, USA). The cells were cultured in a humidified incubator with 5% CO_2_ at 37 °C in DMEM medium (PAN-Biotech, Aidenbach, Germany), supplemented with 10% fetal bovine serum (FBS) and 1% penicillin–streptomycin (pen–strep) solution. These reagents were purchased from Millipore Sigma (Merck KGaA, Darmstadt, Germany). The medium was changed every 2–3 days.

#### 2.4.2. Cell Viability Assays

Cell viability was assessed using the MTT (Thiazolyl blue tetrazolium bromide; cat. no. M5655; Sigma-Aldrich; Merck KGaA, Darmstadt, Germany) assay in 96-well plates. For the assay, 5000 cells in 200 µL of medium were seeded per well and incubated for 24 h at 37 °C. Subsequently, cells were treated with CDs at concentrations of 0.1, 0.8, 1.0, 1.5, 1.8, and 2.35 g/L. Control wells were treated with 0.1% H_2_O and 0.1% DMSO (vehicle controls). After 24 h and 72 h of incubation at 37 °C, the media were aspirated and 100 µL of MTT solution (0.5 mg/mL in PBS) was added to each well. Following 3 h of incubation at 37 °C in the dark, the MTT solution was removed, and 100 µL of DMSO was added to solubilize the formazan crystals. Absorbance was measured using a Tecan Infinite M200 plate reader (Tecan Group Ltd., Männedorf, Switzerland) at 570 nm. Cell viability was calculated relative to the absorbance of vehicle controls. Average values were delivered from three independent experiments (*n* = 3).

#### 2.4.3. Morphological Analysis

Changes in cell morphology were evaluated after cell treatment using a Leica DMI 6000B microscope equipped with a Leica DFC350 FX camera (Leica Microsystems, Wetzlar, Germany). Images were analyzed with the Leica LAS X imaging software (v3.7.4) (Leica Microsystems, Wetzlar, Germany).

#### 2.4.4. Synergistic Effect Analysis

To evaluate the interaction between the chemotherapeutic drug and each carbon dot, we employed the Chou–Talalay method using the CompuSyn software (version 1.0; ComboSyn, Paramus, NJ, USA) to calculate the Combination Index (CI). CI values help classify the interactions: CI < 1 signifies synergism, CI = 1 denotes an additive effect, and CI > 1 indicates antagonism. These simulations were conducted using a non-fixed dose ratio, maintaining a constant concentration of the chemotherapeutic drug while varying the concentrations of each carbon dot.

#### 2.4.5. Statistical Analysis

Cell viability graphs were obtained using the software GraphPad Prism 9 (GraphPad Software Inc., San Diego, CA, USA) after conducting three independent experiments (*n* = 3). Results are represented as the mean ± SD for *n* experiments performed. Statistical analysis was performed with one-way ANOVA tests by Dunnett’s multiple comparisons between the control and treatment groups. Results were considered statistically significant for *p* values < 0.05.

## 3. Results and Discussion

### 3.1. Characterization of CDs

**Figure 1:**
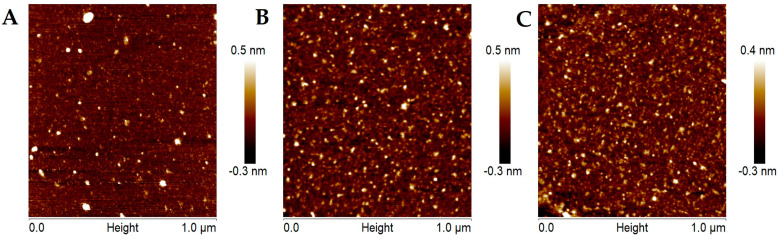
AFM images of CD−1 (**A**), CD−2 (**B**), CD−3 (**C**).

In the current study, four distinct CDs were synthesized using a hydrothermal methodology. Specifically, citric acid and corn stover were used as the carbon precursors (for CD-4, only citric acid was used), and EDA was used to achieve N-doping. The reaction times were set at 4 and 16 h for CD-1 and CD-2, respectively, and 24 h for CD-3 and CD-4. Size distributions were determined through AFM measurements (Figure 1). CD-1, CD-2, and CD-3 presented sizes of ~1 nm, typical of CDs. CD-4 was previously found to present a particle size of ~2 nm [55].

**Figure 2:**
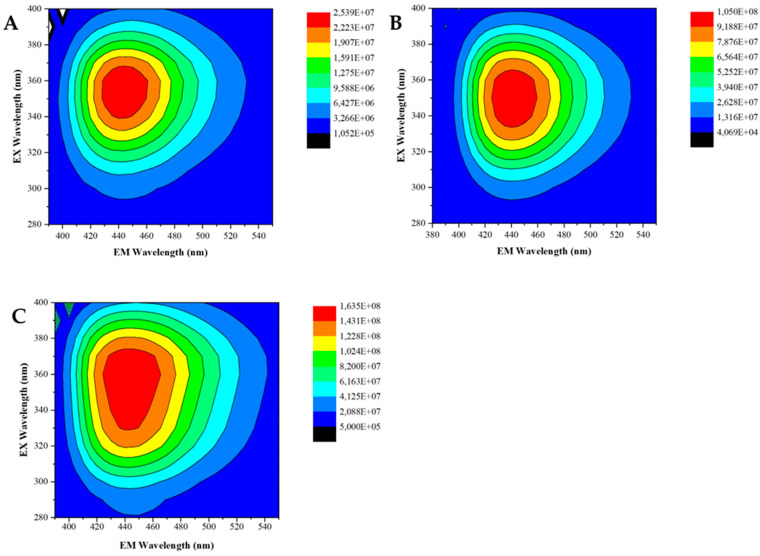
2D fluorescence excitation–emission matrices of CD−1 (**A**), CD−2 (**B**), and CD−3 (**C**).

**Figure 3:**
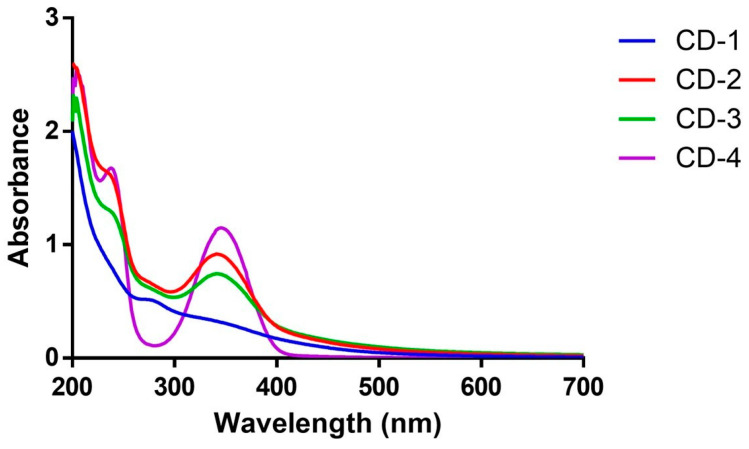
UV–Vis spectra of CDs.

To assess the photoluminescent properties of CDs, 2D excitation–emission contour plots (EECP) were measured (Figure 2), demonstrating similar fluorescent properties between samples. Consistently with the previous analysis [55], CD-1/2/3 exhibited a single emissive center featuring an emission peak at 440 nm. The excitation maxima for these CDs are at ~350 nm. Additionally, we calculated the quantum yield of photoluminescence (QY_PL_) using quinine sulfate as a reference [56]. The resulting values were 9.9%, 38.9%, and 33.1% for CD-1, CD-2, and CD-3, respectively. The absorption spectra of these CDs were also obtained (Figure 3). The results indicate that all three CDs share identical absorption spectra, characterized by a primary band at ~340 nm and a shoulder at ~230 nm. These features are attributed to n-π* and π-π* transitions, respectively [57,58,59]. These findings align with the previous features obtained for CDs [8,33,34,46,60], as well as our previous study of CD-4 [60]. These results are expected as the newly developed CDs are variations of CD-4, using corn stover in the synthesis process.

To gain insights into the ionization state of the nanoparticle surface, we measured the Zeta Potential of these CDs, yielding the following values: −5.9 ± 0.31, −6.3 ± 0.32, −4.2 ± 0.33, and −5.7 ± 0.35 mV for CD-1 to CD-4, respectively. These values collectively suggest that all four CDs display a negative Zeta Potential with no significant variations between them [49,50,52,61].

**Figure 4:**
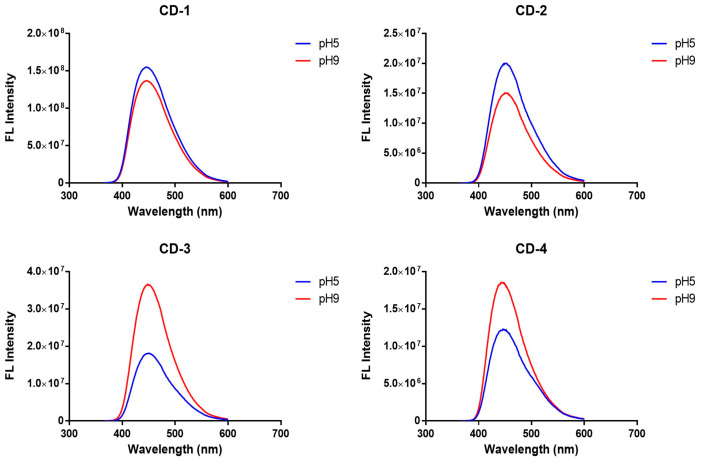
Emission spectra of CDs at pH 5 and 9, in aqueous solution.

To understand the potential pH effect on the fluorescence of CDs, we have measured their emission spectra at pH 5 and 9 (Figure 4). The obtained results show that for all the CDs, either acidic or basic pH present the same maximum emission. More specifically, the maximum emission is ≈440 nm, irrespective of the pH of the media. Given this, all the CDs appear to be stable to pH variations.

**Figure 5:**
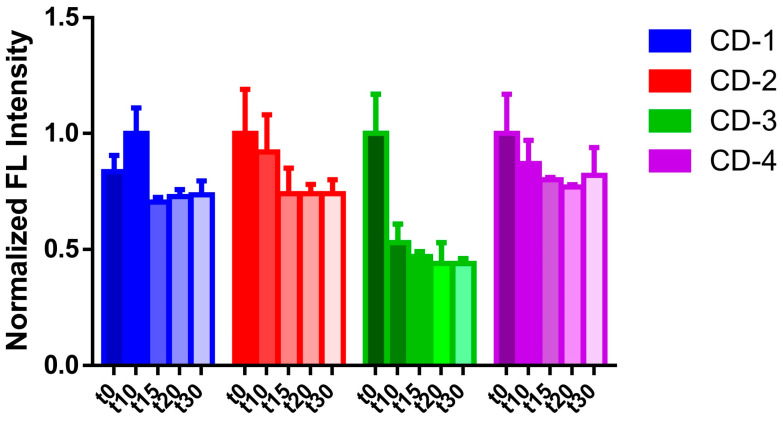
Normalized emission intensity of CDs under UV light irradiation, at t = 0, 10, 15, 20, and 30 min.

We have also evaluated the photostability of CDs, under 30 min UV irradiation, by using an LED light source of 365 nm. More specifically, the photostability was monitored by measuring the emission spectra at t = 0, 10, 15, 20, and 30 min (Figure 5). For CD-1, there was a small increase in emission intensity after 10 min of irradiation, followed by a decrease in emission intensity onward. Nevertheless, this decrease (compared with t = 0 min) appears less relevant. A similar decrease was also seen for CD-2, with the difference being the continuous decrease in emission intensity over time. On the contrary, CD-3 was found to be less photostable, with a decrease of emission intensity to ~50% over 30 min of irradiation, when compared with the values measured at t = 0 min. Finally, CD-4 presents a similar behavior to that of CD-1 and CD-2. Namely, a small decrease in emission intensity over 10/20 min of irradiation, followed by relative stabilization. Thus, CD-1, CD-2, and CD-4 appear to be relatively photostable, with CD-3 showing a significant reduction in emission intensity due to UV light irradiation.

### 3.2. Cytotoxic Effect of CDs on A549 and UM-UC-5 Cell Lines

**Figure 6:**
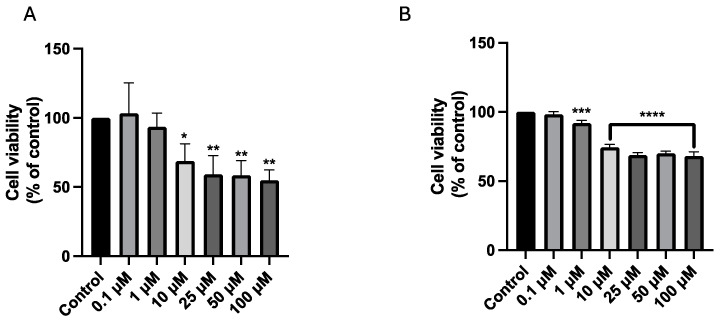
Cell viability of A549 (**A**) and UM-UC-5 (**B**) cancer cells treated with 5-FU for 48 h. Cultured cells were seeded in 96-well plates and treated with 5-FU (0.1–100 µM). Cells treated with vehicle (0.1% DMSO) were used as the control. Values are expressed as percentages of control and represent means ± SD. Each experiment was done three times independently (*n* = 3). * Statistically significant vs. control at *p* < 0.1. ** Statistically significant vs. control at *p* < 0.01. *** Statistically significant vs. control at *p* < 0.001. **** Statistically significant vs. control at *p* < 0.0001.

In our investigation to ascertain the suitability of these nanoparticles for applications in bioimaging and drug delivery, we assessed their potential effects when interacting with cancer cells. Specifically, we studied the cytotoxic effects of the four CDs on A549 and UM-UC-5 cancer cell lines. The cells were incubated with CDs within a 0.1–3.14 g/L concentration range for 24 and 72 h. When choosing these incubation times, we wanted to evaluate the long-term safety of these nanomaterials and ensure the absence of any associated cytotoxic effects. Additionally, we tested the impact of a standard chemotherapeutic agent, 5-fluorouracil (5-FU, Figure 6), and calculated IC50 for future combinations. To evaluate cell viability, we conducted the MTT assay. In this assessment, the control well signifies 100% viable cells, and the percentage of cell viability in each well is expressed relative to that of the control.

The IC50 value serves as a quantitative indicator of the drug’s effectiveness in inhibiting cancer cell growth. Notably, 5-FU demonstrate significant cytotoxic effect to A549 cells above 10 μM, with only 2.42 μM of 5-FU necessary to reduce 50% of A549 cells viability (Figure 6A). UM-UC-5 cells exhibited a modest response to the cytotoxic effects of 5-FU, with concentrations above 1 μM exhibiting significant anti-cancer effects (Figure 6B). Consequently, a concentration of 2.42 and 4.21 µM of 5-FU was applied to A549 and UM-UC-5 cells, respectively, for further experimentation.

**Figure 7:**
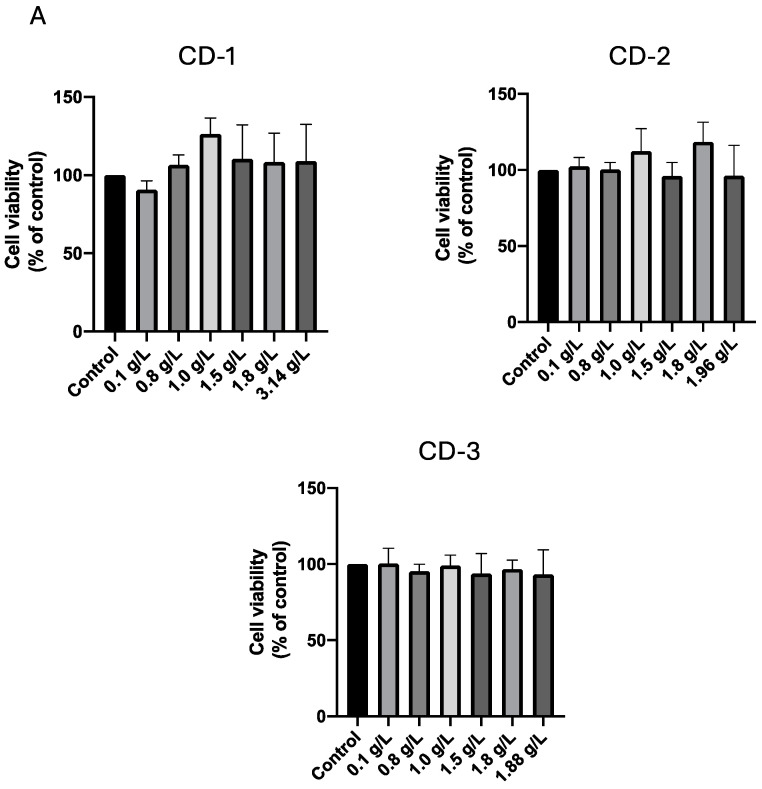

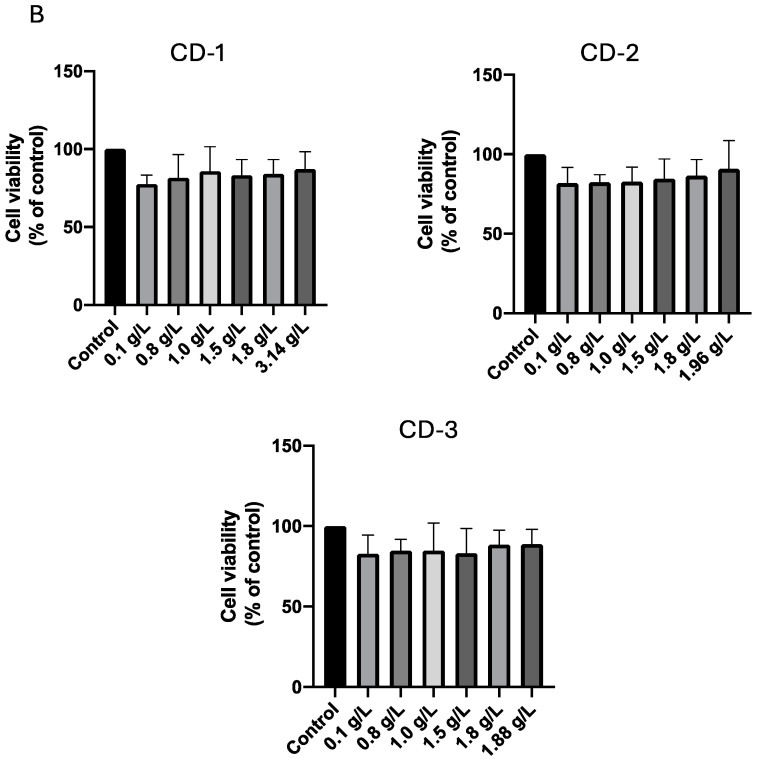
Cell viability of A549 (**A**) and UM-UC-5 (**B**) cancer cells treated with CD-1–3 for 24 h. Cultured cells were seeded in 96-well plates and treated with increasing concentrations of CD-1–3. Cells treated with vehicle (0.1% DMSO) were used as the control. Values are expressed as percentages of control and represent means ± SD. Each experiment was done three times independently (*n* = 3).

The results of the 24-h incubation period (Figure 7) indicate that, at this stage, the CDs exerted no discernible effect on either A549 (Figure 7A) or UM-UC-5 (Figure 7B) cancer cells. Cellular viability remained consistent across the tested concentrations. Notably, our team’s previous evaluation of CD-4 on A549 cancer cells [55] also revealed no significant cytotoxic effects after 24 and 72 h of incubation. Thus, the cytotoxic effect of CD-1/2/3 in these cancer cells is consistent with previous results for CD-4. Curiously, CD-1 and CD-2 induced a significant increase in cell viability that could be attributed to either an elevated cell count in the well or increased metabolic activity of the cells. Variations in cellular metabolism or minor pipetting errors leading to slightly higher cell seeding in specific wells can result in samples exceeding 100% viability, which is considered normal. Our findings indicate that these CDs may also impact cell growth, as confirmed by cell counting with trypan blue.

**Figure 8:**
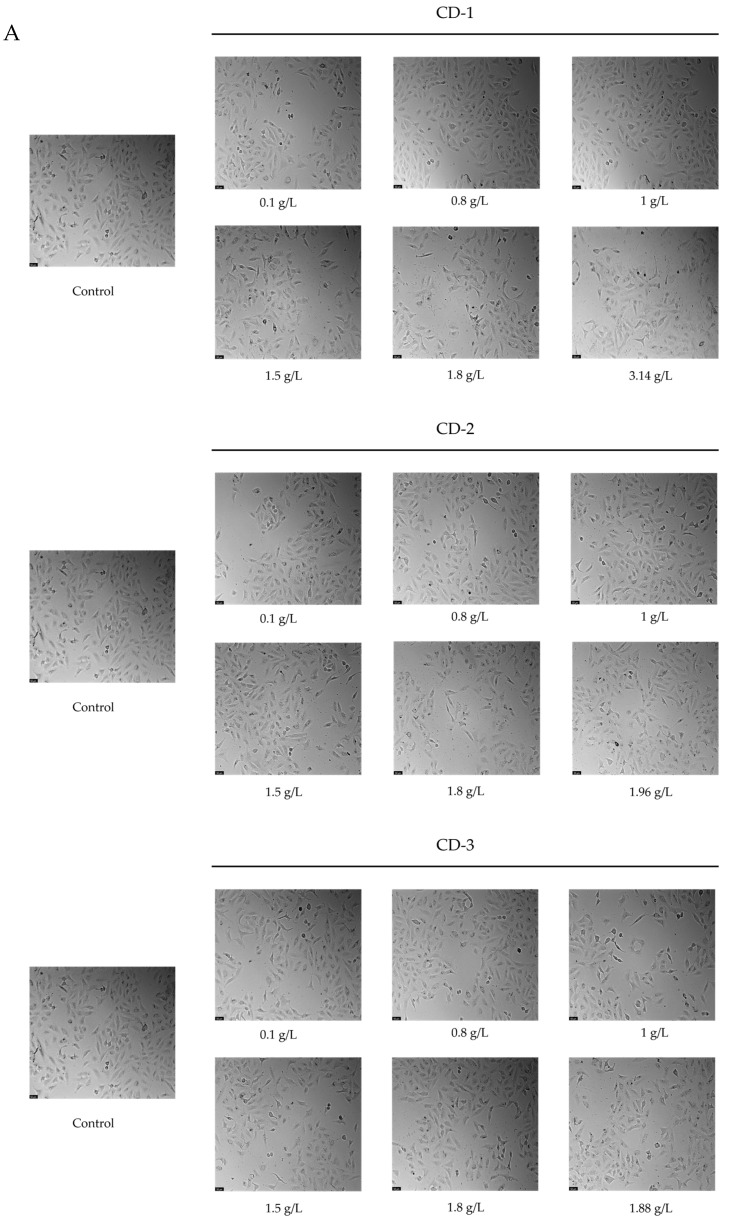

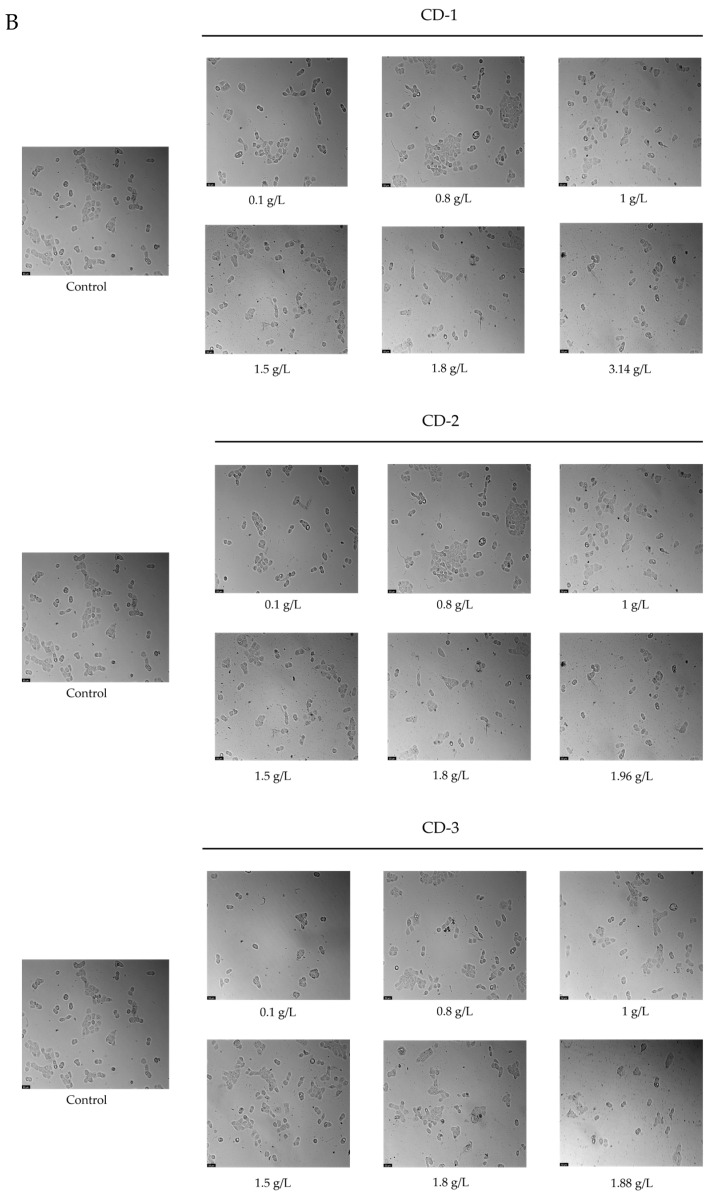
Morphological analysis of A549 (**A**) and UM-UC-5 (**B**) cancer cells treated with CDs for 24 h of incubation.

Morphological evaluations were conducted to examine potential modifications in the phenotype of both A549 and UM-UC-5 cells (Figure 8). Consistently with cellular viability, at the 24-h incubation mark, no significant morphological alterations were detected for the tested nanoparticles. This observation suggests that, within this timeframe, the nanoparticles did not induce noticeable changes in the visual appearance or structure of the studied cancer cells. These findings collectively suggest that, within the investigated timeframe, the CDs did not induce significant cytotoxicity in the studied cancer cell lines.

**Figure 9:**
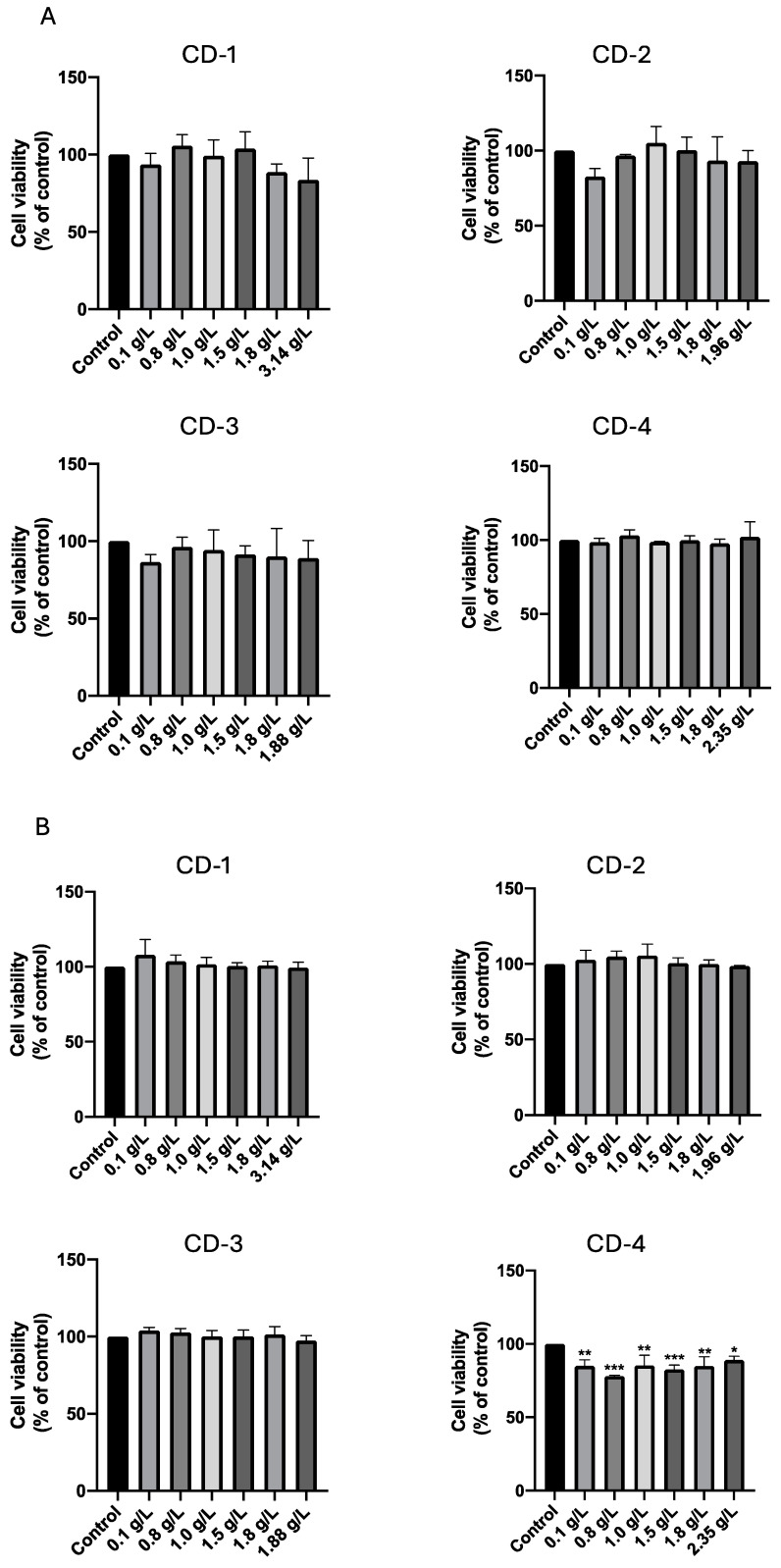
Cell viability of A549 (**A**) and UM-UC-5 (**B**) cancer cells treated with CD-1–4 for 72 h. Cultured cells were seeded in 96-well plates and treated with increasing concentrations of CD-1–4. Cells treated with vehicle (0.1% DMSO) were used as the control. Values are expressed as percentages of control and represent means ± SD. Each experiment was done three times independently (*n* = 3). * Statistically significant vs. control at *p* < 0.1. ** Statistically significant vs. control at *p* < 0.01. *** Statistically significant vs. control at *p* < 0.001.

Then, we extended the incubation time of this analysis to a period of 72 h when accessing the cell viability of A549 and UM-UC-5 cancer cells in the presence of CDs. The results (Figure 9) indicate that no effect was observed on cell viability for the A549 cancer cell line across the range of CD concentrations studied (Figure 9A). On the other hand, when analyzing the results for the UM-UC-5 cancer cell line (Figure 9B), CD-4 induced a decrease in cell viability across all studied concentrations.

Unlike CD-1, CD-2, and CD-3, the results suggest that CD-4 may not be suitable for potential use in UM-UC-5 cancer cell lines due to its impact on cell viability. However, its application in A549 cancer cells appears to be fitting.

In addition, we report here a class of CDs, obtained from a synthesis that is simple and uses organic waste as precursors. These CDs present a safety profile for both A549 and UM-UC-5 cells for future biological applications at different times of incubation.

**Figure 10:**
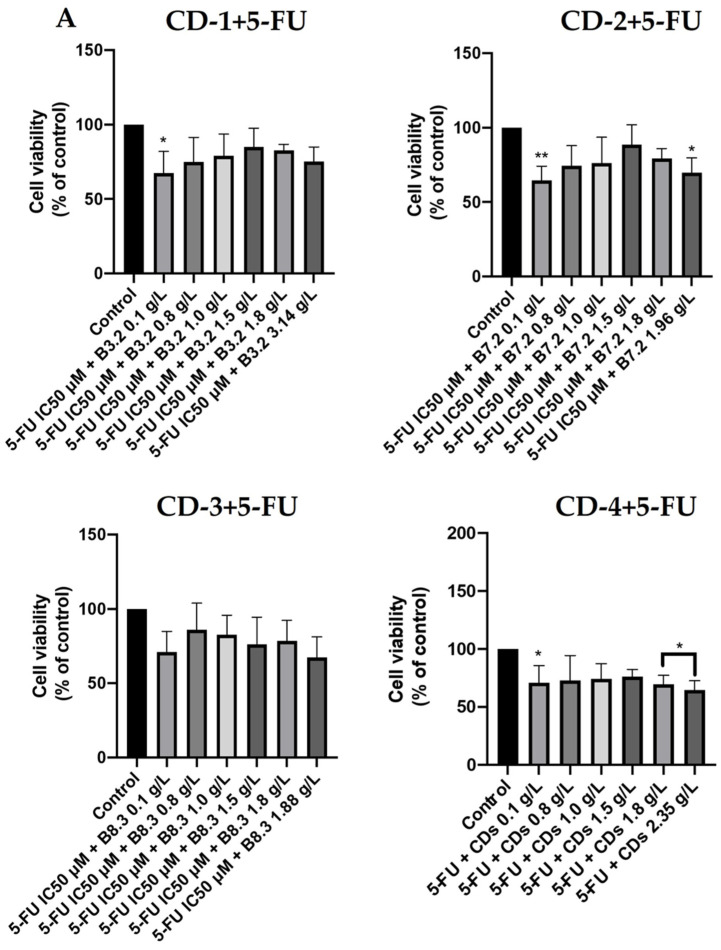

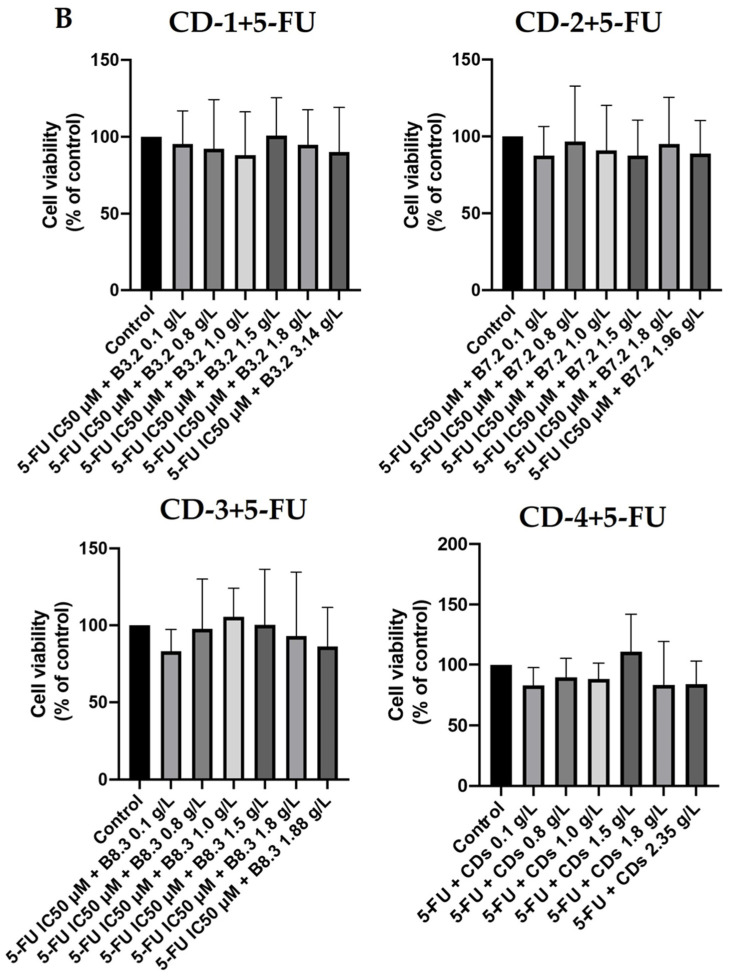
Cell viability of A549 (**A**) and UM-UC-5 (**B**) cancer cells treated with CD-1–4 (0.1–3.14 g/L) in combination with IC50 of 5-FU (4.21 µM for UM-UC-5 and 2.42 µM for A549 cells) for 24 h of incubation. Cells treated with vehicle (0.1% DMSO) were used as the control. Values are expressed as percentages of control and represent means ± SD. Each experiment was done three times independently (*n* = 3). * Statistically significant vs. control at *p* < 0.1. ** Statistically significant vs. control at *p* < 0.01.

**Figure 11:**
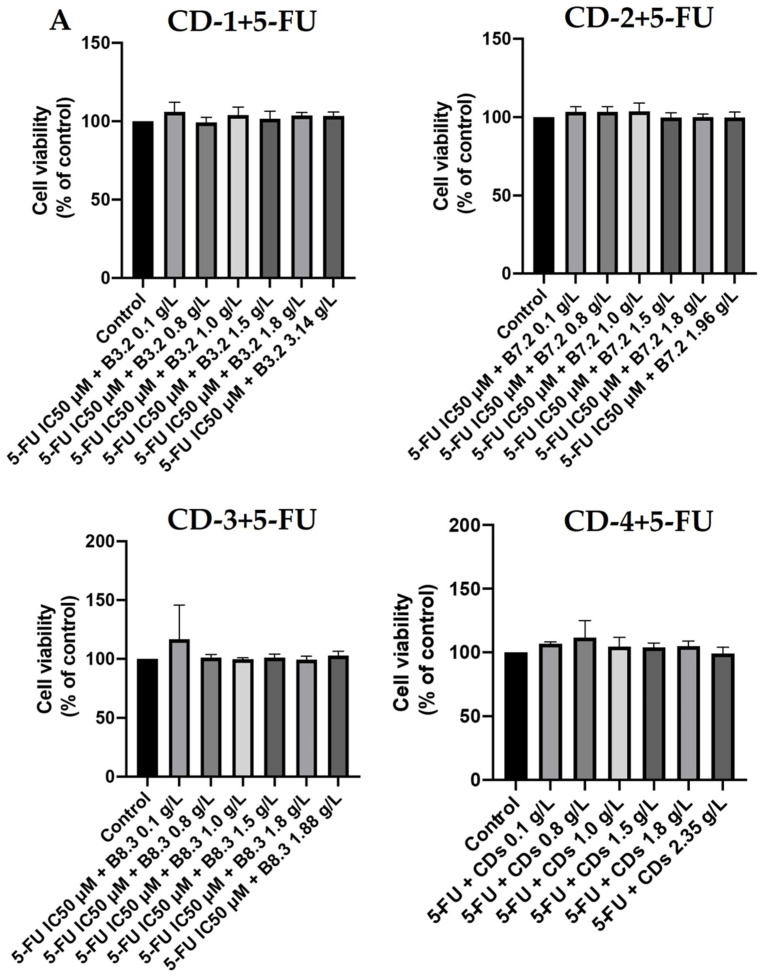

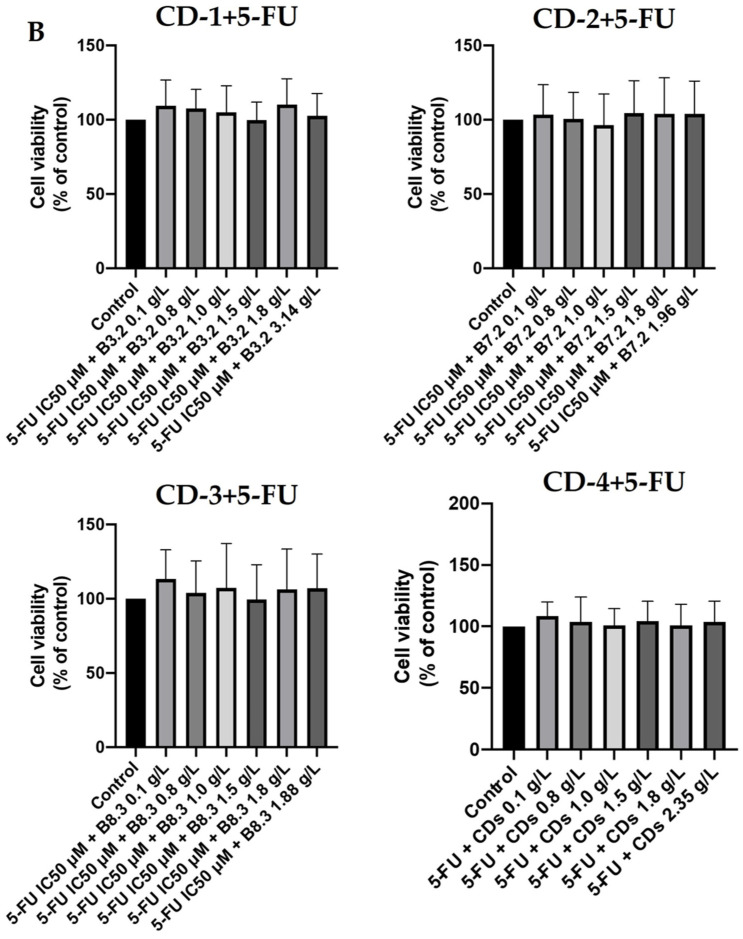
Cell viability of A549 (**A**) and UM-UC-5 (**B**) cancer cells treated with CD-1–4 (0.1–3.14 g/L) in combination with IC50 of 5-FU (4.21 µM for UM-UC-5 and 2.42 µM for A549 cells) for 72 h of incubation. Cells treated with vehicle (0.1% DMSO) were used as the control. Values are expressed as percentages of control and represent means ± SD. Each experiment was done three times independently (*n* = 3).

To gain further insights into the potential applicability of these CDs in drug delivery, we investigated their behavior in the presence of a drug used in cancer treatment, 5-FU [52,53]. We assessed the cell viability of A549 and UM-UC-5 cancer cells with increasing concentrations of CDs (0.1–3.14 g/L), in combination with the IC50 of 5-FU, for each cancer cell line (4.21 µM for UM-UC-5 and 2.42 µM for A549 cells) over 24 and 72 h of incubation. As depicted in Figure 10A, the combination of CD-1, CD-2, and CD-4 with 5-FU only decreased A549 cell viability at a CD concentration of 0.1 g/L. Similar effects were also observed for higher concentrations of CD-2 (1.96 g/L) and CD-4 (1.80 and 2.35 g/L). In contrast, for UM-UC-5 cells (Figure 10B), CDs in combination with 5-FU showed no significant impact on cell viability. Interestingly, with a 72-h incubation period (Figure 11), none of the CDs affected cell viability for both cancer cell lines. These findings suggest that the CDs are protecting the cells and mitigating the effects of 5-FU. This phenomenon could be explained by the CDs’ ability to interact with the cells in a way that reduces the cytotoxicity of 5-FU [61,62]. The CDs might be facilitating a mechanism of cellular resistance or altering the uptake of 5-FU by the cancer cells. Another possibility is that the CDs possess antioxidant properties that help protect the cells from the adverse effects of 5-FU [63]. This means that these CDs are not suitable for acting as drug carriers for this type of drug, as they mitigate its therapeutic effect. However, they could be of use in potential applications in which there is a need to mitigate the toxic effects of chemotherapeutic agents.

### 3.3. Combination Index Evaluation in the Combination of Carbon Dots CD-1–CD-4 with 5-FU in A549 and UM-UC-5 Cancer Cell Lines

**Figure 12:**
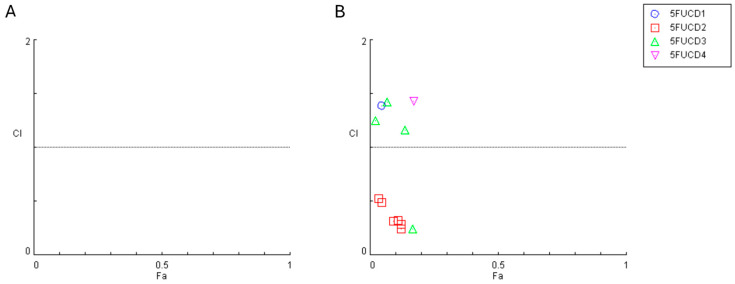
Graphical representation of combination index plot obtained from the CompuSyn Report for the combinations of IC50 5-FU with increasing concentrations of carbon dots 1–4, obtained using the Chou–Talalay method for (**A**) A549 cell line and (**B**) UM-UC-5 cell line for 24 h.

**Figure 13:**
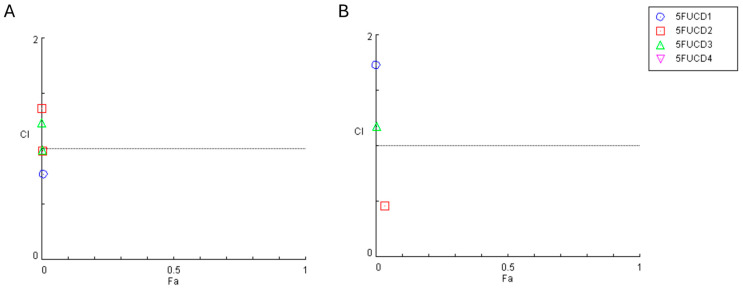
Graphical representation of combination index plot obtained from the CompuSyn Report for the combinations of IC50 5-FU with increasing concentrations of carbon dots 1–4, obtained using the Chou–Talalay method for (**A**) A549 cell line and (**B**) UM-UC-5 cell line for 72 h.

To explore the effects of combining the antineoplastic agent 5-FU with CD-1–CD-4 in A549 and UM-UC-5 cancer cell lines, we employed the Chou–Talalay method to calculate the combination index (CI). The CI plot for the combinations of 5-FU with CD-1–4 over 24 h and 72 h are shown in Figure 12 and Figure 13, respectively and illustrate the CI values against the fractional effect (Fa). CI values above one indicates antagonism, values equal to one indicate additivity, and values below one indicate synergism. A Fa value of zero indicates no cell death, whereas a Fa value of one indicates complete cell death. These results are summarized in Table 1.

The combination index plot obtained for the combinations in A549 for 24 h demonstrates that all CI values obtained are above one, which indicates that all combinations present antagonistic interactions. Regarding the UM-UC-5 cell line, the results for the combination of 5-FU with CD-1, 3, and 4 show the same results as above. However, the combination with CD-2 shows synergistic interactions at all concentrations.

After 72 h of incubation, the combination index plot obtained for the combinations in A549 and the results for the A549 cell line remain similar, since almost all the combinations present antagonistic interactions (Table 2). In fact, there are two combinations (1.5 g/L CD-2 and 1.8 g/L CD-3) that come very close to an additive interaction (CI = 1). Regarding the UM-UC-5 cell line, the results for the combination of 5-FU with CDs present antagonistic interactions at all combinations, except for the CD-2 at a concentration of 1 g/L.

## 4. Conclusions

Recent research on carbon dots (CDs) has highlighted their potential as valuable assets for applications in bioimaging and drug delivery, among others. Considering this, in this study, we successfully synthesized four distinct N-doped CDs using a hydrothermal methodology. In addition, CDs were obtained from a “green” synthesis that is simple and uses organic waste as a precursor. In our investigation into the applicability of these nanoparticles for bioimaging and drug delivery, we examined their potential cytotoxic effects on A549 and UM-UC-5 cell lines. The 24-h incubation period showed no discernible effects on cellular viability, indicating that the CDs did not induce cytotoxicity within this timeframe. Morphological evaluations at 24 h revealed no notable changes in cell phenotype. Extended incubation periods of 72 h demonstrated that CD-4 might not be suitable for UM-UC-5 cancer cells due to its effects on cell viability and morphology. In contrast, all four nanoparticles proved promising for applications in A549 cancer cells, emphasizing their potential in different contexts using these cancer cells. Further exploration of combination assays with a chemotherapeutic drug (5-FU) revealed that the CDs protect the cells and mitigate the toxic effects of this drug. It was also observed that most CDs presented antagonistic interaction with 5-FU, except CD-2, which presented synergistic interaction, opening a new strategy for future studies in which CDs can be employed as chemoprotective agents.

## Figures and Tables

**Table 1:** Fa and CI values of carbon dots and 5-FU (2.42 μM for A549 or 4.21 for UM-UC-5) combinations for 24 h in A549 and UM-UC-5 cells. CI in bold indicates drug pairs that are synergic and addictive.

Cell Line	Carbon Dot (g/L)	CD-1		CD-2		CD-3		CD-4	
A549		Fa	CI	Fa	CI	Fa	CI	Fa	CI
	0.1	0.32571	>10	0.35358	4.07950	0.29050	>10	0.29327	>10
	0.8	0.25045	>10	0.25625	>10	0.14106	>10	0.27112	>10
	1.0	0.21080	>10	0.23775	>10	0.17399	>10	0.25960	>10
	1.5	0.14973	>10	0.11464	>10	0.24125	>10	0.23729	>10
	1.8	0.17149	>10	0.20749	>10	0.21533	>10	0.30515	>10
	3.14/1.96/1.88/2.35	0.24841	2.5 × 10^7^	0.30239	>10	0.32533	>10	0.35487	2.0 × 10^7^
UM-UC-5	0.1	0.04691	1.38739	0.12282	**0.24168**	0.16829	**0.24545**	0.17125	1.42858
	0.8	0.07871	9.49265	0.03349	**0.52381**	0.02238	1.25008	0.10344	6.23997
	1.0	0.12014	>10	0.09244	**0.31672**	1.0 × 10^−6^	>10	0.11796	8.72487
	1.5	1.0x10^−6^	>10	0.12338	**0.28694**	1.0 × 10^−6^	>10	1.0 × 10^−6^	>10
	1.8	0.05181	>10	0.04826	**0.48553**	0.06963	1.42030	0.16593	>10
	3.14/1.96/1.88/2.35	0.09961	>10	0.11033	**0.32097**	0.13757	1.16018	0.16034	>10

**Table 2:** Fractional effect (Fa) and CI values of carbon dots and 5-FU (2.42 μM for A549 or 4.21 for UM-UC-5) combinations for 72 h in A549 and UM-UC-5 cells. CI in bold indicates drug pairs that are synergic and addictive.

Cell Line	Carbon Dot (g/L)	CD-1		CD-2		CD-3		CD-4	
A549		Fa	CI	Fa	CI	Fa	CI	Fa	CI
	0.1	1.0 × 10^−6^	>10	1.0 × 10^−6^	>10	1.0 × 10^−6^	>10	1.0 × 10^−6^	>10
	0.8	0.00689	**0.76924**	1.0 × 10^−6^	>10	1.0 × 10^−6^	>10	1.0 × 10^−6^	>10
	1.0	1.0 × 10^−6^	>10	1.0 × 10^−6^	>10	0.00228	1.23695	1.0 × 10^−6^	>10
	1.5	1.0 × 10^−6^	>10	0.00368	**0.98253**	1.0 × 10^−6^	>10	1.0 × 10^−6^	>10
	1.8	1.0 × 10^−6^	>10	1.0 × 10^−6^	>10	0.00365	**0.99106**	1.0 × 10^−6^	>10
	3.14/1.96/1.88/2.35	1.0 × 10^−6^	>10	0.00187	1.36813	1.0 × 10^−6^	>10	0.00760	>10
UM-UC-5	0.1	1.0 × 10^−6^	>10	1.0 × 10^−6^	>10	1.0 × 10^−6^	>10	1.0 × 10^−6^	>10
	0.8	1.0 × 10^−6^	>10	1.0 × 10^−6^	>10	1.0 × 10^−6^	>10	1.0 × 10^−6^	>10
	1.0	1.0 × 10^−6^	>10	0.03624	**0.45725**	1.0 × 10^−6^	>10	1.0 × 10^−6^	>10
	1.5	0.00255	1.73160	1.0 × 10^−6^	>10	0.00554	1.17674	1.0 × 10^−6^	>10
	1.8	1.0 × 10^−6^	>10	1.0 × 10^−6^	>10	1.0 × 10^−6^	>10	1.0 × 10^−6^	>10
	3.14/1.96/1.88/2.35	1.0 × 10^−6^	>10	1.0 × 10^−6^	>10	1.0 × 10^−6^	>10	1.0 × 10^−6^	>10

## Data Availability

The original contributions presented in the study are included in the article, further inquiries can be directed to the corresponding author/s.
